# 

**Published:** 1869-06-01

**Authors:** 


### CONTENTS.

REPORTS.

American Medical Association—Session for 1869 .......... 333

Illinois State Medical Society .......... 347

Chicago Medical Society .......... 359

Editorial .......... 363

Obituary .......... 363

Original Communications .......... 364

